# Haptoglobin and Sickle Cell Polymorphisms and Risk of Active Trachoma in Gambian Children

**DOI:** 10.1371/journal.pone.0011075

**Published:** 2010-06-11

**Authors:** Mathilde Savy, Branwen J. Hennig, Conor P. Doherty, Anthony J. Fulford, Robin Bailey, Martin J. Holland, Giorgio Sirugo, Kirk A. Rockett, Dominic P. Kwiatkowski, Andrew M. Prentice, Sharon E. Cox

**Affiliations:** 1 MRC International Nutrition Group, London School of Hygiene & Tropical Medicine, London, United Kingdom; 2 MRC Laboratories, Fajara, The Gambia; 3 Department of Infectious Tropical Diseases, London School of Hygiene & Tropical Medicine, London, United Kingdom; 4 Department of Medical Genetics, San Pietro Hospital, Rome, Italy; 5 Wellcome Trust Centre for Human Genetics, Oxford, United Kingdom; 6 Malaria Programme, Wellcome Trust Sanger Institute, Hinxton, United Kingdom; University of Montreal, Canada

## Abstract

**Background:**

Susceptibility and resistance to trachoma, the leading infectious cause of blindness, have been associated with a range of host genetic factors. *In vitro* studies of the causative organism, *Chlamydia trachomatis*, demonstrate that iron availability regulates its growth, suggesting that host genes involved in regulating iron status and/or availability may modulate the risk of trachoma. The objective was to investigate whether haptoglobin (Hp) haplotypes constructed from the functional polymorphism (Hp1/Hp2) plus the functional promoter SNPs -61A-C (rs5471) and -101C-G (rs5470), or sickle cell trait (HbAS, rs334) were associated with risk of active trachoma when stratified by age and sex, in rural Gambian children.

**Methodology and Principal Findings:**

In two cross sectional surveys of children aged 6–78 months (n = 836), the prevalence of the clinical signs of active trachoma was 21.4%. Within boys, haplotype E (-101G, -61A, Hp1), containing the variant allele of the -101C-G promoter SNP, was associated with a two-fold increased risk of active trachoma (OR = 2.0 [1.17–3.44]). Within girls, an opposite association was non-significant (OR = 0.58 [0.32–1.04]; P = 0.07) and the interaction by sex was statistically significant (P = 0.001). There was no association between trachoma and HbAS.

**Conclusions:**

These data indicate that genetic variation in Hp may affect susceptibility to active trachoma differentially by sex in The Gambia.

## Introduction

Trachoma, a chronic keratoconjunctivitis caused by the intracellular bacterium *Chlamydia trachomatis*, is the leading infectious cause of blindness worldwide [1]. Repeated episodes of infection cause intense conjunctival inflammation (active trachoma), which can lead to chronic infection and conjunctival scarring, corneal scarring, opacification and, ultimately, blindness [2]. Trachoma disease progression is variable and incidence varies by age and sex [3], [4]. Some individuals experience prolonged infection, while others clear their infections in a few weeks. Differences in the inflammatory response to infection have been proposed as a determinant of progressive disease [5], [6], [7]. Although interventions have considerably reduced the number of people with blinding trachoma over the past decades, current estimates indicate that active trachoma still affects some 80 million people worldwide and about 8 million people are visually impaired [8].

Host genetic factors play a major role in susceptibility or resistance to many infectious diseases [9], [10], including *C. trachomatis* [11], [12]. Genetic association studies so far, have focussed on trachoma scarring or trachomatous trichiasis and human polymorphisms within loci involved in immunity and inflammation [13] and include several within the TNF locus including the -308G-A TNF-α promoter single nucleotide polymorphism (SNP) [14], [15]; the -1082A-G IL-10 SNP [16], [17]; chemokine and cytokine clusters in chromosomes 4q & 5q31 and HLA class I alleles [18], reviewed in [12]. Active trachoma in Gambian children has also been associated with the IL-10 -3917-G allele [19]. Acquisition of iron is a fundamental step in the development of a pathogen within its host. Evidence from *in vitro* studies suggests that low iron availability leads to impaired growth and infectivity of *C. trachomatis* [20], [21] but may also contribute to persistence or re-activation of dormant infection [20], [22], [23], whilst the anti-chlamydial action of some compounds is reversed in the presence of an iron source including holo-transferrin [24]. The accumulation of transferrin and its receptors around chlamydial inclusions in low-iron environments may suggest the mechanism of iron acquisition by *C. trachomatis* [20], [25]. In combination these and other studies suggest that variation in host genes involved in modulating immune, particularly inflammatory type responses and in regulating iron status and/or iron availability may affect the outcome of trachoma infection.

Haptoglobin (Hp), an acute phase protein, encoded by two major co-dominant alleles, Hp1 and Hp2, results in three functionally distinct phenotypes, Hp11, Hp12 and Hp22. Promoter polymorphisms -61A-C (rs5471) and -101C-G (rs5470) have been associated with ahaptoglobinaemia and hypohaptoglobinaemia, respectively [26], whilst reporter gene assays also demonstrated that -61C promoter constructs had significantly decreased transcriptional activity [27]. Haptoglobin binds circulating, toxic, free hemoglobin (Hb) released during intravascular haemolysis – such as occurs during malarial infections. The resultant complex is taken up by CD163 on circulating monocytes and macrophages leading both to an altered cytokine secretory profile [28], [29] and the eventual recycling of the iron component of haem for erythropoiesis. Plasma concentrations of haptoglobin, and the binding affinities of Hp for free hemoglobin and of the Hp-Hb complex for CD163 vary by phenotype. Hp22 has been associated with evidence of increased oxidant stress and iron delocalisation [30], [31], [32], as well as an increased risk of malaria-associated anemia [33], [34]. Hp is also thought to affect immune regulation, including the balance of Th1∶Th2 cytokine responses [35] and several studies have associated haptoglobin phenotypes and genotypes with a range of conditions including cardiovascular disease [36], diabetes [37], [38], HIV infection [39], [40], [41] and susceptibility to malaria [42], [43], [44], [45] including a protective association of the Hp haplotype containing the Hp2 and the C allele for the promoter -61A-C (rs5471) SNP in Gambian children [46]. A protective effect of the genetic variant sickle cell trait (HbAS) against malaria has been well documented [47], [48], [49], [50], [51]. However, few studies have assessed whether HbAS may be protective against other conditions [50]. There is evidence that alpha thalasaemia, which is protective against severe malaria, is also protective against severe morbidity from other infections, perhaps due to an interaction between malaria and risk of other infections [52].

Therefore we investigated whether Hp haplotypes constructed from the functional Hp allele (Hp1 or Hp2) plus the functional promoter SNPs -61A-C (rs5471) and -101C-G (rs5470) and HbAS were associated with the risk of active trachoma in Gambian children.

## Methods

### Design and sampling

Two cross sectional surveys of clinical signs of acute trachoma of children aged 6–78 months were conducted in September 2003 & January 2005 in eight rural Mandinka villages in the West Kiang district of The Gambia. As some children were visited in both years and others only once, only the first visit of each child was considered in the analysis. Children known to have sickle cell disease (HbSS) (n = 4) were excluded from analysis.

### Grading of *Chlamydia trachomatis* infection

Clinical signs of active trachoma were assessed using a ×2 binocular loupe and pen torch by single grader. Both eyelids were everted and scored according to the WHO simplified trachoma grading system [53]. Active trachoma was defined as the presence of grades TF (follicular trachoma) or TI (intense inflammatory trachoma) in one or both eyes, with the eye with the greatest clinical severity being used for the outcome result. Eight children were found to have trachomatous scarring (TS) but none with trichiasis (TT). These eight children were excluded from the current analysis for risk of active trachoma. All children with active trachoma were offered treatment with tetracycline eye ointment as per Gambian national eye care programme guidelines at the time. Finally, following further structured surveys as part of a national programme [54], these communities were treated with 3 annual rounds of oral azithromycin, completed in 2009 as per WHO and the International Council of Ophthalmology guidelines for community control strategies.

### Genotyping and haplotype construction

DNA was extracted from peripheral blood leukocytes using a standard salting out method [55]. Aliquots of DNA were shipped to the Wellcome Trust Centre for Human Genetics, Oxford, UK, where all genotyping was conducted. Haptoglobin was genotyped by allele-specific PCR adapted from a method published by Koch et al. [56]. This method determines the Hp1 and Hp2 alleles but does not distinguish between the “F” and “S” subtypes of the Hp1 and Hp2 alleles. It therefore avoids potential misclassification based on the presence of these sub-types in different populations [57]. PCR products were resolved by agarose gel electrophoresis and visualised under UV light. Details of primers and cycling conditions are provided in the supplementary material (Table S1). Sequenom® MassARRAY® (Sequenom®, Hamburg Germany) was used to genotype the haptoglobin promoter -61A-C (rs5471), -101C-G (rs5470) SNPs and HbS (rs334: hemoglobin – sickle) according to manufacturer's instructions.

Genotyping accuracy was verified by sequencing (eurofins MWG operon) in both directions in 56 samples with different Hp haplotypes. PCR products for sequencing were generated using primers Hp-Ex-U (5′-GCA GTG TGA AAA TCC TCC AAG ATA A-3′) and Hp-Ex1-L (5′-AAT TTA GCC CAT TTG CCC GTT TCT T-3′) under standard conditions.

Haplotypes for the two Hp promoter SNPs and the Hp gene alleles (Hp1 and Hp2) were constructed using SNPHAP [58].

### Ethical permission

The study was approved by the joint Medical Research Council/Gambian Government Ethics Committee. The Gambian National DNA Collection Guidelines were followed regarding the handling of genetic material and information [59]. Parental written informed consent was obtained for all study participants.

### Data analyses

Data analyses were performed using SAS version 9.1 (SAS Institute, Cary NC, USA). Differences in the prevalence of active trachoma between villages and by age (in years) and sex of children were tested using logistic regression. We have previously observed age-dependent effects of Hp variation on malaria risk [46] [45] within a sub-set of these children, whilst iron status can differ by sex in young children [60]. Therefore we analysed associations between genotypes and active trachoma using logistic regression models stratified by age group (greater or less than the median age of 36 months, to reduce the number of groups tested and increase precision of the estimates of effect) and by sex, and subsequently tested for interactions by comparison of models with and without inclusion of the interaction terms gene*age (greater or less than 36 months) or gene*sex in models. Village was included in all models *a priori*, whilst in models stratified by age, sex was included *a priori* and age (in years) was included a priori in models stratified by sex. Hp haplotypes were fitted as categorical variable with 0, 1 or 2 copies. As children who were found to be HbSS were excluded from analyses, sickle genotype was fitted as a simple binary variable (HbAS vs. HbAA).

## Results

### Demographic and genetic characteristics of study subjects

A total of 1155 children were examined for clinical signs of trachoma. Of these, 235 (20.3%) had active trachoma, 11 (0.95%) had signs of trachomatous inflammation (TI) and 8 (0.69%) with trachomatous scarring (TS). There was no evidence of a difference by sex (**Table 1**). DNA samples were available from 1089 of these children. Complete genetic, clinical and socio-demographic data were available for 836 children with complete Hp haplotypes, and for 807 children screened for HbS and were included in the current analyses. The mean age of children included in the analysis was 35.6±18.1 months. The distribution of covariates and genotypes for children included in the analysis versus those not included is presented in **Table 1**. With the exception of village of residence (P = 0.01) there were no statistically significant difference in the distribution of covariates between the included/excluded groups, including the prevalence of active trachoma. There were no differences in the distribution of genotypes between the two groups, except for the Hp-61A-C promoter SNP for which fewer AA homozygotes and more heterozygotes were detected in the children included in the analysis (P = 0.02). Genotype frequencies did not differ by village of residence, except for HbAS (P = 0.0004) (data not shown).

**Table 1:** Characteristics of the study population.

	n	Children included in the analysis [%]	n	Children not included in the analysis [%]^a^	P-value
*Sociodemographic variables*					
**Age (month)**	836		282		
< = 36		54.1		52.1	NS
>36		45.9		47.9	
**Age (year)**	836		282		
1		35.4		34.7	NS
2		18.4		17.4	
3		17.7		15.6	
4		15.4		14.2	
5		13.0		18.1	
**Sex**	836		317		
male		50.4		51.1	NS
female		49.6		48.9	
**Village**	836		317		
a		10.0		7.3	0.01
b		11.7		12.0	
c		23.3		15.8	
d		8.8		10.4	
e		12.9		11.7	
f		12.4		18.6	
g		9.0		8.5	
h		11.7		15.8	
*Genotype distribution*					
**No of copies of Hp2 allele**	836		61		
0		20.9		21.3	NS
1		58.0		59.0	
2		21.1		19.7	
**-101C-G**	836		162		
CC		73.2		71.0	NS
CG		25.4		25.3	
GG		1.4		3.7	
**-61A-C**	836		161		
AA		74.8		83.2	0.02
AC		25.2		16.8	
CC		0.0		0.0	
**HbS**	807		177		
HbAA		82.0		86.4	NS
HbAS		18.0		13.7	
*Trachoma status*					
**Active trachoma** a	836		317		
Yes		21.4		17.7	NS
No		78.6		82.3	

a Out of the 1155 children who had trachoma assessment, 836 children were included in the final analyses and 319 were not (because of missing data or because they were found to have sickle cell disease (HbSS), trachomatous scarring (TS) or protein-energy malnutrition as determined by weight-for-height z-score<−3 SD). In the 836 individuals included HWE p-values were <0.0001; 0.184, and <0.0001 for Hp1/2, -101C-G, and -61A-C, respectively.

Genotypes were not in Hardy Weinberg equilibrium **(** **Table 1** **)** for Hp1/2 as well as the -61A-C polymorphism, for which, as previously reported [46], no CC homozygotes were detected. Sequencing 56 samples in both directions confirmed the absence of -61C-C homozygotes and genotypes determined by sequencing correlated 100% with those determined by hME Sequenom typing for both Hp promoter SNPs.

Four common (frequency >10%) and two less common (frequency ≤1%) Hp haplotypes were detected **(** **Table 2** **)**. As previously reported [26] the variant -61C allele appeared much more frequently in conjunction with the Hp2 allele (haplotype D), whilst the -101G allele was found more often with the Hp1 allele (haplotype E). No haplotypes containing the variant alleles for both of the promoter SNPs were detected.

**Table 2:** Prevalence of haplotypes constructed from main haptoglobin alleles (Hp1 and Hp2) and Hp promoter SNPs -101C-G (rs5470) and-61A-C (rs5471).

Haplotype	-101C-G (rs5470)	-61A-C (rs5471)	Hp	Haplotype frequency (n = 2*836)	Copy number of haplotypes N = 836, n (%)		
					0	1	2
A	C	A	1	36%	321 (38.4)	431 (51.5)	84 (10.0)
B	C	A	2	37%	319 (38.2)	408 (48.8)	109 (13.0)
C	C	C	1	0.5%	832 (99.5)	4 (0.5)	0 (0.0)
D	C	C	2	12%	629 (75.2)	207 (24.7)	0 (0.0)
E	G	A	1	14%	614 (73.4)	212 (25.4)	10 (1.2)
F	G	A	2	0.5%	832 (99.5)	4 (0.4)	0 (0.0)

### Associations between active trachoma and haptoglobin haplotypes and sickle cell trait

The prevalence of active trachoma did not vary by age or sex of the study participants, but did by village of residence, ranging from 7.1 to 34.7% (P = 0.0004) **(** **Table 3** **)**.

**Table 3:** Active trachoma as a function of children age, sex and village of residence included in the current analysis (n = 836).

	n	Active Trachoma (%)	OR [CI]	P-value
**Age (mo)**				
< = 36	452	21.0	1.0	NS
>36	384	21.9	1.05 [0.75–1.46]	
**Age (year)**				
1	296	18.6	1.0	NS
2	154	25.3	1.49 [0.93–2.37]	
3	148	25.0	1.46 [0.90–2.34]	
4	129	19.4	1.05 [0.62–1.78]	
5	109	21.1	1.17 [0.68–2.02]	
**Sex**				
female	421	22.1	1.0	NS
male	415	20.7	0.92 [0.66–1.28]	
**Village**				
a	84	7.1	1.0	**0.0004**
b	98	19.4	3.13 [1.18–8.24]	
c	195	23.1	3.90 [1.59–9.54]	
d	74	16.2	2.52 [ 0.89–7.08]	
e	108	25.9	4.55 [1.78–11.59]	
f	104	15.4	2.36 [0.88–6.34]	
g	75	25.3	4.41 [ 1.65–11.75]	
h	98	34.7	6.91 [ 2.73–17.48]	

When stratified by sex. The Hp haplotype E (-101G, -61A, Hp1), containing the variant allele of the -101C-G promoter SNP, was associated with a two-fold increased risk of active trachoma in boys, but with a non-significantly decreased risk in girls (P = 0.07) (**Table 4**). This interaction was statistically significant (P = 0.001). There was no evidence of an association of the prevalence of active trachoma with the other Hp haplotypes or with HbAS vs HbAA, in either sex or either age group (data not shown by age group).

**Table 4:** Associations between active trachoma and haplotypes or genotypes by sex.

	Females (n = 421^a^)			Males (n = 415^a^)			*P-value interaction sex*
	n	%	OR [0.95 CI]	n	%	OR [0.95 CI]	
**Haplotype (No of copies)**							
A							
0	159	21.8	1.0	162	22.7	1.0	NS
1	216	19.1	0.84 [0.51–1.40]	215	15.4	0.62 [0.36–1.05]	
2	46	20.7	0.93 [0.41–2.13]	38	21.5	0.93 [0.39–2.23]	
*P-value*		NS			NS		
B							
0	169	18.5	1.0	150	19.3	1.0	NS
1	202	19.9	1.10 [0.64–1.85]	206	18.5	0.94 [0.55–1.62]	
2	50	27.9	1.70 [0.80–3.61]	59	18.6	0.95 [0.44–2.07]	
*P-value*		NS			NS		
D^b^							
0	312	18.6	1.0	317	19.6	1.0	NS
1	109	25.2	1.47 [0.86–2.51]	98	16.0	0.78 [0.42–1.44]	
2	0	-	-	0	-	-	
*P-value*		NS			NS		
E^b^							
0	305	22.8	1.0	309	15.9	1.0	***0.001***
1	110	14.6	0.58 [0.32–1.04]	102	27.5	2.0 [1.17–3.44]	
2	6	-	-	4	-	-	
*P-value*		0.07			0.04		
**Genotype**							
HbAA	334	20.2	1.0	328	17.8	1.0	NS
HbAS	76	19.1	0.93 [0.49–1.75]	69	22.9	1.37 [0.71–2.64]	
*P-value*		NS			NS		

Analyses by sex are adjusted for age in years and village.

a except for the analysis with HbS where n = 410 for the girls only group, n = 397 for the boys only group.

b Due to small numbers individuals with 2 copies of these haplotypes were not included in the analysis.

## Discussion

The prevalence of active trachoma was high, an average of 20%, compared to 6% in a smaller survey of 140 children aged 1–9 years in West Kiang District, conducted in 2006 [54]. The prevalence of active trachoma varied significantly by village, similar to other observations in The Gambia [54]. We did not find any difference in the rate of active trachoma between age groups or sex, contrary to some reports in which older children (3–5 years old) and females are more likely to have trachoma [3], [4]. However, it is unclear how much of previously observed sex and age differences relate to inherent responses to infection or to cultural practices leading to differences in exposure [4].

Here we report a sex-specific effect of the Hp haplotype E (-101G, -61A, Hp1) and susceptibility to active trachoma in Gambian children. The small number of individuals with two copies of the Hp haplotype E did not allow us to assess whether there might be a dose effect of carriage of this haplotype on risk of trachoma. In 257 Gambian children with no malaria infection, haplotype E (-101G, -61A, Hp1) was highly associated with a decreased risk of having non-detectable levels of plasma Hp (OR = 0.41, p = 0.002) [46]. It is possible that an effect of this Hp haplotype on risk of active trachoma is mediated though increased levels of Hp and differential inflammatory responses from interactions between Hp1 or Hp2 protein with macrophages [35], T cells [61], or other immune-modulatory effects [62] including possible effects on adaptive immunity [63]. We cannot currently explain why the effects of this Hp haplotype differ by sex; however, sex differences in disease patterns and in responses to vaccination are well known, and it is increasingly recognised that many immune functions may differ by sex even in young children [64], [65]. Sex specific effects of Hp phenotype and markers of iron status have been documented in some [66], but not other studies [67] for reasons that are not clear. Data on iron status was available for a small number of individuals (N<250), preventing us from carrying out meaningful analysis of possible interactions between Hp haplotype and iron status.

As we have previously reported in a sub-set of this population [46], we did not detect any C-C homozygotes for Hp -61A-C. To confirm that the absence of -61C-C homozygotes was not due to technical issues the accuracy of the genotyping method for the two Hp promoter SNPs was confirmed by sequencing 56 samples, selected to represent each haplotype. Indeed we have replicated these results again employing another hME Sequenom assay employing different primers taking into account two other promoter polymorphisms in the same vicinity (rs5469 3bp from -101C-G and rs5472 6pb from -61A-C; unpublished data, Rockett & Cox). The distribution of the Hp haplotypes reported by ourselves previously was confirmed in this larger sample [46], whilst the associations between the promoter SNPs with the Hp1 & Hp2 alleles has also been previously reported in West Africans from Ghana [26]. Similarly, publicly available data through the National Center for Biotechnology Information (NCBI http://www.ncbi.nlm.nih.gov/) indicates that -61C-C homozygotes are not present in populations of Caucasian, Pacific RIM, Hispanic, African American and African ancestry, with exception of the Yoruba panel where 1 out of 24 individuals was found to be homozygous for the C allele. This supports our earlier hypothesis that this polymorphism is under some selection pressure (similar to HbS), possibly from a deleterious effect of carriage of two copies of the C allele, resulting in the absence of -61C-C homozygotes.

In summary, in a cohort of 836 children up to 6.5 years of age across 8 villages in West Kiang The Gambia, the prevalence of active trachoma was 21.4%. Our observation of an increased risk of active trachoma in children with the Hp haplotype (-101G, -61A, Hp1), in boys, with a non-significant decreased risk in girls and statistically significant interaction warrants further investigation of possible associations between Hp polymorphisms and inflammatory, immune and infectious outcomes by sex.

## Supporting Information

Table S1 Haptoglobin Hp1/Hp2 - PCR conditions and primer sequences - method adapted from Koch et al. 2003.
(0.05 MB DOC)
