# Supplementary material for: Haptoglobin and Sickle Cell Polymorphisms and Risk of Active Trachoma in Gambian Children
Source: PLoS One. 2010 Jun 11;5(6):e11075. doi: 10.1371/journal.pone.0011075 (PMC2884021; doi:10.1371/journal.pone.0011075)
Supplement: Table S1 — Haptoglobin Hp1/Hp2 - PCR conditions and primer sequences - method adapted from Koch et al. 2003. (0.05 MB DOC) [file pone.0011075.s001.doc]

Table S1 Haptoglobin Hp1/Hp2 - PCR conditions and primer sequences - method adapted from Koch et al. 2003

| **reaction mix for Hp2 allele** | **ul per reaction** | **final concentrations/ amounts** | **reaction mix for Hp1 allele** | **ul per reaction** | **final concentrations/ amounts** |
| --- | --- | --- | --- | --- | --- |
| **PE buffer x 10** | 1 | x1 | **PE buffer x 10** | 1 | x1 |
| **MgCl2 25 mM** | 0.8 | 1.5 uM | **MgCl2 25 mM** | 0.8 | 1.5 uM |
| **dNTPs 8 mM** | 0.25 | 200 umol | **dNTPs 8 mM** | 0.25 | 200 umol |
| **Hp-Koch Primer mix C & D (both 5 uM)** | 0.6 | 0.3uM | **Hp-Koch Primer mix A & B (both 5 uM)** | 0.6 | 0.3uM |
| **Control primer mix "T" (F&R) 5uM** | 0.3 | 0.15 uM | **Control primer mix "A"(F&R) 5uM** | 0.3 | 0.15 uM |
| **TAQ GOLD 5U/ul** | 0.1 | 0.5U/ul | **TAQ GOLD 5U/ul** | 0.1 | 0.5U/ul |
| **H20 millipore 18.2** | 1.95 |  | **H20 millipore 18.2** | 1.95 |  |
| **Total reaction mix** | 5 |  | **Total reaction mix** |  |  |
| **DNA 1 ng/ul** | 5 | 5 ng | **DNA 1 ng/ul** | 5 | 5 ng |
| **total reaction mix + DNA/well** | **10** |  | **total reaction mix + DNA/well** | **10** |  |
|  |  |  |  |  |  |
| **Cycling parameters** |  |  | **Gel** = 1% Agarose, 220mV, aprox 75-90 mins |  |  |
| temp | time |  |  |  |  |
| 95 | 14 min |  | AB = 1757bp | Hp1 allele product | |
| 95 | 30sec | | | CD = 349 bp | Hp2 allele product | |
| 69 | 30sec | | x 35 | A = aprox 1100 bp | control product | |
| 72 | 2min | | | T =aprox 659 bp | control product | |
|  |  |  |  |  |  |
| 72 | 7 min |  |  |  |  |
| **Primers** |  |  |  |  |  |
| Primer A | F | 5'-GAG GGG AGC TTG CCT TTC CAT TG-3' | |  |  |
| Primer B | R | 5'-GAG ATT TTT GAG CCC TGG CTG GT-3' | |  |  |
| Primer C | F | 5'-CCT GCC TCG TAT TAA CTG CAC CAT-3' | |  |  |
| Primer D | R | 5'-CCG AGT GCT CCA CAT AGC CAT GT-3' | |  |  |
|  | F | 5' ACA CAG AAC CCA CGG ACA CA-3' | |  |  |
| Control Primer A | R | 5' AGG CGC TAA CCA GGA TGT TC-3' | |  |  |
|  | F | 5' GAG AAA GGC CCA GAC TCC AAG CAT C-3' | |  |  |
| Control Primer T | R | 5' GTG TAG ACC TGA CCA GCG AGT GTG-3' | |  |  |
